# The anisotropic network model web server at 2015 (ANM 2.0)

**DOI:** 10.1093/bioinformatics/btu847

**Published:** 2015-01-06

**Authors:** Eran Eyal, Gengkon Lum, Ivet Bahar

**Affiliations:** ^1^Cancer Research Institute, Sheba Medical Center, 2 Sheba Rd, Ramat Gan 52621, Israel and ^2^Department of Computational and System Biology, University of Pittsburgh, 3501 Fifth Ave, Pittsburgh, PA 15213, USA

## Abstract

**Summary:** The anisotropic network model (ANM) is one of the simplest yet powerful tools for exploring protein dynamics. Its main utility is to predict and visualize the collective motions of large complexes and assemblies near their equilibrium structures. The ANM server, introduced by us in 2006 helped making this tool more accessible to non-sophisticated users. We now provide a new version (ANM 2.0), which allows inclusion of nucleic acids and ligands in the network model and thus enables the investigation of the collective motions of protein–DNA/RNA and –ligand systems. The new version offers the flexibility of defining the system nodes and the interaction types and cutoffs. It also includes extensive improvements in hardware, software and graphical interfaces.

**Availability and implementation:** ANM 2.0 is available at http://anm.csb.pitt.edu

**Contact:** eran.eyal@sheba.health.gov.il, eyal.eran@gmail.com

## 1 Introduction

The last decade has seen a remarkable increase in the number of studies that explore biomolecular systems dynamics using coarse-grained normal mode analysis, prompted by the introduction and establishment of elastic network models (ENMs). ENMs are efficient and accurate frameworks for robust predictions of cooperative, often functional, movements under equilibrium conditions (Bahar *et al*., 2010). More recently the low frequency modes predicted by ENMs were further shown to be useful in applications that extend beyond equilibrium motions, such as efficient sampling of conformational space, mapping of trajectories between known states, structure refinement and molecular docking (see, e.g. Das *et al*., 2014; Eyal *et al*., 2011; Hu *et al*., 2013; Leis and Zacharias, 2011; Meireles *et al*., 2011; Ostermeir and Zacharias, 2014; Peng *et al*., 2010; Rueda *et al*., 2009; Schröder *et al*., 2007). A series of web servers have been developed to assist in the evaluation of normal modes either at atomic level [e.g. MoViES (Cao *et al*., 2004), NOMAD-Ref (Lindahl *et al*., 2006) and NMSim (Krüger *et al*., 2012)], or at a coarse-grained level, often using ENMs [e.g. ElNémo (Suhre and Sanejouand, 2004), WEBnm@ (Hollup *et al*., 2005), iGNM (Yang *et al*., 2005), AD-ENM (http://enm.lobos.nih.gov), and Promode Elastic (Wako and Endo, 2011)].

Among ENMs, the anisotropic network model (ANM) and similar residue-based spring-and-node models, introduced 15 years ago (Atilgan *et al.*, 2001; Doruker *et al.*, 2000; Hinsen, 1998; Sanejouand and Tama, 2001), have found wide applications in molecular and structural biology due to their simplicity, yet proved successful for predicting the directions of large-scale functional motions in accord with experimental observations (Bahar *et al*., 2011; Bakan *et al*., 2009). Despite the simple theory behind the model, applying the code and exploring the results require some knowledge in command line scripting and molecular graphics. The ANM server, we introduced in 2006 (Eyal *et al.*, 2006) was also intended to fill this gap for users with little background in computational biology. It essentially allowed exploration of the dynamics in one button click—upon inputting the name of the Protein Data Bank (PDB) file (or coordinate files in PDB format) of the structure of interest.

Nine years later, we now present an improved version, ANM 2.0, of the server, which includes, alongside with hardware and software upgrades, significant improvement in the functionality and capabilities of the site, in particular the extension from proteins-only to biomolecular complexes and assemblies with DNA, RNA and ligands.

## 2 The ANM 2.0 interface

**Fig. 1.**
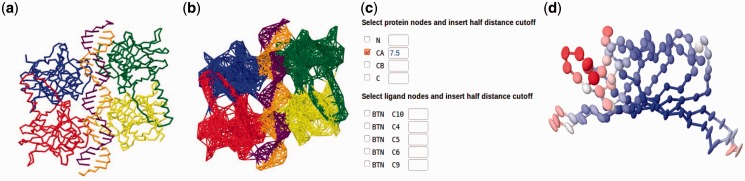
Some of the new features of ANM 2.0 web server: The new version is now applicable to nucleotide-containing structures. A snapshot of p53 bound to DNA (PDB 3Q05) is displayed by backbone stick representation (**a**) as well as network representation (**b**). Each nucleotide is represented by three nodes in this default setting, with a uniform 15Ȧ cutoff distance. (**c**) Versatility of the server for defining network nodes and assigning atom-specific interaction ranges. **(d**) Interactive representation of anisotropic ADPs as color-coded ellipsoids, supported by Jmol version 13.0.16. The ADPs computed for streptavidin (PDB 1STP) are shown. This PDB file contains no experimental data on ADPs

The most important new feature is the option to now construct network models for structures which include nucleotides and small molecule/ligands, and thus visualize the collective dynamics of broader range of bio-molecular structures (Fig. 1a and b). By default each nucleotide is represented in the network by three nodes, positioned at the P atom of the phosphate group, C4′ in the base and C2 in the sugar ring. This model has been shown (Yang *et al.*, 2006) to have superior predictive abilities, based on the comparison with experimental B-factors using 64 oligonucleotide/protein complexes, and on case studies such as the application of ANM to describe ribosomal dynamics (Wang *et al*., 2004). The server additionally provides the option of adopting different numbers of user-defined atoms for representing the nucleotide nodes. Small ligands can also be explicitly included in the ANM analysis. Ligands, if present in the PDB coordinates file, will be automatically parsed and mapped by the server into network nodes (Fig. 1c).

To support the above changes, the server applies a new strategy that enables flexibility in the definition of network nodes and cutoff distances of interaction. The cutoff distance of interaction, *r*_c_, now depends on the identity of interacting atoms. For each atom type *i*, an associated distance range of interaction, *t*_i_, is assigned. For each pair of atom types *i, j* the cutoff distance *r*_c_ is defined as *t*_i_*+t*_j_. This definition implies that the number of parameters in the system is bound by the number of different atom types. In the simplest case where we wish a unified threshold distance *r*_c_ for all atom types, as in the traditional ANM, we simply assign *t*_i_* = r*_c_ */2* for all atom types we would like to include. The server suggests *r*_c_* = 15 *Å by default, as a compromise between better agreement with isotropic B-factors, obtained in larger cutoffs (Eyal *et al*., 2006) and more realistic anisotropic displacement parameters (ADPs) obtained using lower cutoffs (Eyal *et al*., 2007). The selection of specific and non-standard nodes is technically done using the ‘advanced input’ page, accessible from the ANM home page, following the selection of a structure file.

Another important improvement is the addition of Matlab (MathWorks) as the method of choice for eigenvalue decomposition for a subset of normal modes. We use the *eigs* function which is the Matlab interface to the ARPACK package (Lehoucq *et al*., 1998) to evaluate a selected subset of eigenvalues and eigenvectors from a sparse matrix. This allows now to change the number of modes to be calculated online. More modes than the (earlier) default number of 20 can be evaluated and visualized if needed. Conversely, fewer modes can also be requested, especially for large systems, to increase efficiency. Blzpack (http://crd-legacy.lbl.gov/∼osni/#Software), the eigenvalue decomposer of ANM version 1.0 can still be used to calculate a fix number (20) of modes as an alternative method.

The server now produces .*xyz* coordinates file to support the visualization of vibrations along different directions in Jmol. This format is simpler and more widely used than the Gamess file which was in use in the original ANM server. Gamess file is still produced by the new server and can be downloaded. The new ANM server also enjoys the many new capabilities developed by Jmol (http://www.jmol.org) molecular graphics team since the previous version. Most notably and related to ANM, Jmol supports now interactive visualization of ADPs as ellipsoids framing the spatial location of atoms within a defined percentile of the Gaussian distribution. ADPs are a byproduct of ANM calculations (Eyal *et al*., 2007) and we now offer such interactive representation in the ADP part of the results. The server also presents static images produced by Raster3D (Merritt and Bacon, 1997). A convenient option to take screenshots and save them locally in jpg format was added, as saving images directly from the applet is not yet supported. Other improvements in the graphical user interfaces, such as the more convenient navigation in the correlation maps and the inter-residue distance fluctuation maps permit a direct assessment of structural regions subject to distinctive cross-correlations as well as those that are highly decoupled.

In the new version, the user has the additional option of importing external data, e.g. submitting the principal modes that have been calculated with another method, and take advantage of the easy and powerful GUI of the server for visualizing the modes of motions and the cross-correlations between residue motions. The dimensions of these uploaded vectors must match the number of system nodes defined by the user.

Finally, the new server now enjoys 128 GB of RAM which allows for better performance for analyzing the collective motions of large molecules. Software versions currently being used are: Matlab R2014a, Jmol 13.0.16, Raster3D 3.02. Most of the envelope and cgi code is written in Perl 5.18. We also added the core C and Matlab code for inclusion of nucleic acids and ligand atoms into the source distribution packages available at: http://anm.csb.pitt.edu/anmdocs/source.html

## Funding

This work was sponsored by NIH Awards 1R01GM099738 and 5R01GM086238.

*Conflict of Interest*: none declared.
